# Tuberculosis Preventive Therapy among Persons Living with HIV, Uganda, 2016–2022

**DOI:** 10.3201/eid2903.221353

**Published:** 2023-03

**Authors:** Deus Lukoye, Gail Gustavson, Proscovia M. Namuwenge, Simon Muchuro, Estella Birabwa, Seyoum Dejene, Julius Ssempiira, Julius N. Kalamya, Steven Baveewo, Odile Ferroussier-Davis, Lisa A. Mills, Emilio Dirlikov, Lisa J. Nelson, Stavia Turyahabwe

**Affiliations:** US Centers for Disease Control and Prevention, Kampala, Uganda (D. Lukoye, G. Gustavson, J. Ssempiira, J.N. Kalamya, S. Baveewo, L.A. Mills, E. Dirlikov, L.J. Nelson);; Uganda Ministry of Health, Kampala (P.M. Namuwenge, S. Muchuro, S. Turyahabwe);; US Department of Defense, Kampala (E. Birabwa);; US Agency for International Development, Kampala (S. Dejene);; US Centers for Disease Control and Prevention, Atlanta, Georgia, USA (O. Ferroussier-Davis)

**Keywords:** tuberculosis and other mycobacteria, bacteria, HIV/AIDS and other retroviruses, respiratory infections, latent tuberculosis infection, tuberculosis preventive therapy, PEPFAR, Uganda

## Abstract

During October 2016–March 2022, Uganda increased tuberculosis (TB) preventive therapy coverage among persons living with HIV from 0.6% to 88.8%. TB notification rates increased from 881.1 to 972.5 per 100,000 persons living with HIV. Timely TB screening, diagnosis, and earlier treatment should remain high priorities for TB/HIV prevention programming.

Tuberculosis (TB) is the leading cause of illness and death globally among persons living with HIV (PLHIV) (*1*,*2*). In 2021, among ≈38.4 million PLHIV worldwide, 703,000 TB cases and 187,000 TB-related deaths were reported (*3*,*4*). HIV antiretroviral therapy (ART) and TB preventive therapy (TPT) reduce TB incidence and death among PLHIV (*5*,*6*). TPT is administered to persons at high risk for TB and who do not have symptoms of active disease; positive tuberculin skin tests or interferon gamma release assays are not required. Although increased ART coverage has coincided with declines in TB-related deaths worldwide, since the early 2000s, TPT scale-up has been limited (*3*,*7*,*8*).

Uganda is a World Health Organization–designated TB and HIV high-burden country (*3*). By 2020, ≈1,400,000 PLHIV were reported in Uganda; in 2021, a total of 29,000 TB cases and 6,200 TB-related deaths among PLHIV were reported (*9*,*10*). In 2015, Uganda accelerated efforts to provide TPT to PLHIV who had no TB symptoms and received support from the US President’s Emergency Plan for AIDS Relief (PEPFAR) (*11*,*12*). Further efforts included a 100-day scale-up campaign in 2019, adopting TPT as standard of care for all eligible PLHIV in 2021 (*13*), and a last-mile campaign launched in June 2022. Therefore, despite the COVID-19 pandemic, TPT scale-up continued. We analyzed data describing TPT scale-up among PLHIV in Uganda and highlight next steps to further reduce TB-related illness and death and reach global targets for treatment coverage.

## The Study

We sourced semiannual aggregate data during October 2016–March 2022 from the centralized PEPFAR DATIM Monitoring, Evaluation, and Reporting database (Uganda DATIM version 1.31 MER 2.5, updated January 4, 2021; https://ug.datim4u.org). We analyzed trends among PLHIV receiving PEPFAR-supported ART across 5 areas: TPT initiation, defined as beginning any TPT regimen, such as 6-month daily isoniazid or 3-month 1 time/week isoniazid plus rifapentine; TPT completion, defined as receiving a full course of TPT according to data capture tools; TPT completion rates, calculated as the number of TPT completions divided by the number of TPT initiations in the previous semiannual period × 100; TPT coverage, calculated as the number of TPT completions divided by the total number of PLHIV eligible for TPT; and TB notification among PLHIV on ART, calculated as the number of registered new and relapsed TB patients with documented HIV-positive status divided by the total number of PLHIV on ART. TPT eligibility was defined as 95% of total PLHIV on ART; TPT ineligibility (5%) accounts for PLHIV with active TB and PLHIV discontinuing TPT because of loss to follow-up, death, or adverse events. Since October 2019, PLHIV have been considered to be on ART until <27 days after their last missed clinical appointment; clients whose last missed appointment was >28 days earlier are not considered to be receiving ART. Before October 2019, PLHIV were considered to be on ART until <90 days after their last missed appointment.

We used R software version 4.1.1 (The R Project for Statistical Computing, https://www.r-project.org) to analyze age groups, sex, and region. We described semiannual trends by using time series plots. We used nonparametric Cuzick tests to analyze TB notification rates across periods.

The total number of PLHIV on ART in Uganda increased from 890,938 in March 2017 to 1,283,662 in September 2022. Most (1,226,266; 95.5%) PLHIV were >15 years of age, and 822,864 (65.5%) were women. Age and sex distribution remained stable over time.

**Figure 1:**
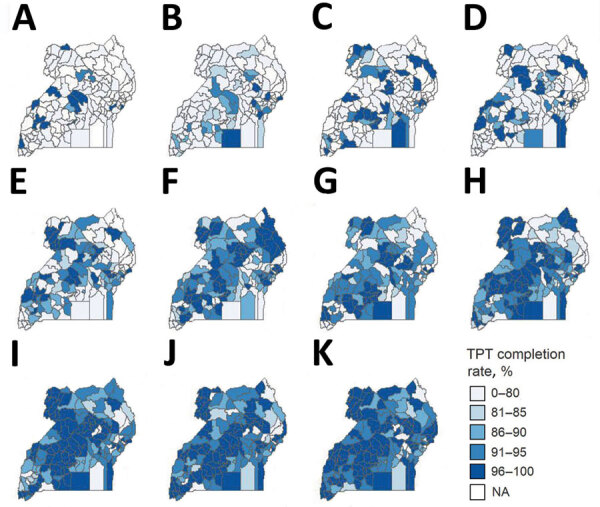
Completion rate percentages for tuberculosis preventive therapy by district among persons living with HIV in Uganda during 2016–2022. Completion rates in different districts in Uganda for 11 semiannual periods: A) October 2016–March 2017; B) April 2017–September 2017; C) October 2017–March 2018; D) April 2018–September 2018; E) October 2018–March 2019; F) April 2019–September 2019; G) October 2019–March 2020; H) April 2020–September 2020; I) October 2020–March 2021; J) April 2021–September 2021; and K) October 2021–March 2022. TPT, tuberculosis preventive therapy.

TPT completions increased from 28.6% (5,264/18,394) during October 2016–March 2017 to 94.0% (75,173/79,949) during October 2021–March 2022 (Table 1; Figure 1). We observed a steady increase in TPT completion rates except for a decline from 71.1% during April–September 2017 to 63.6% during October 2017–March 2018. The average regional TPT completion rate increased from 27.2% (interquartile range [IQR] 12.9%–41.9%) during October 2016–March 2017 to 93.8% (IQR 91.85%–96.25%) during October 2021–March 2022. The lowest regional average completion rate across all periods was 69.9% in the Kigezi region, and the highest average completion rate was 85.2% in the Lango region.

**Table 1:** Tuberculosis preventive therapy completion and coverage among persons living with HIV who received antiretroviral therapy during semiannual periods, Uganda, October 2016–March 2022*

Semiannual periods	Initiated TPT	Completed TPT	Receiving ART†	TPT eligible‡	Cumulative completion	Completion rate, %§	TPT coverage, %¶
2016 Oct–2017 Mar	18,394	5,264	890,938	846,391	5,264	28.6	0.6 (5,264/846,391)
2017 Apr–2017Sep	17,391	12,360	993,070	943,417	17,624	71.1	1.9 (17,624/943,417)
2017 Oct–2018 Mar	8,162	5,188	1,030,756	979,218	22,812	63.6	2.3 (22,812/979,218)
2018 Apr–2018 Sep	18,836	13,272	1,101,716	1,046,630	36,084	70.5	3.4 (36,084/1,046,630)
2018 Oct–2019 Mar	99,602	85,954	1,097,366	1,042,498	122,038	86.3	11.7 (122,038/1,042,498)
2019 Apr–2019 Sep	443,900	391,514	1,148,258	1,090,845	513,552	88.2	47.1 (513,552/1,090,845)
2019 Oct–2020 Mar	111,898	98,399	1,201,166	1,141,108	611,951	87.9	53.6 (611,951/1,141,108)
2020 Apr–2020 Sep	172,862	158,546	1,218,006	1,157,106	770,497	91.7	66.6 (770,497/1,157,106)
2020 Oct–2021 Mar	122,969	113,296	1,239,829	1,177,838	883,793	92.1	79.1 (883,793/1,117,838)
2021 Apr–2021 Sep	134,387	124,525	1,266,588	1,203,259	1,008,318	92.7	83.8 (1,008,318/1,203,259)
2021 Oct–2022 Mar	79,949	75,173	1,283,662	1,219,479	1,083,491	94.0	88.8 (1,083,491/1,219,479)

*Patients received ART at PEPFAR-supported facilities. ART, antiretroviral therapy; PEPFAR, US President’s Emergency Plan for AIDS Relief; TPT, tuberculosis preventive therapy. †Number of adults and children receiving ART was reported quarterly; we used the number reported during the final quarter of each 6-month period. ‡TPT eligible was defined as 95% of total persons living with HIV who were receiving ART; TPT ineligibility (5%) accounts for PLHIV with active TB disease and PLHIV discontinuing TPT because of loss to follow-up, death, or adverse events. §Number of patients who completed TPT divided by the number of patients who initiated TPT × 100. ¶TPT coverage was calculated as cumulative number of persons living with HIV who completed TPT divided by the number of TPT eligible patients ´ 100.

**Figure 2:**
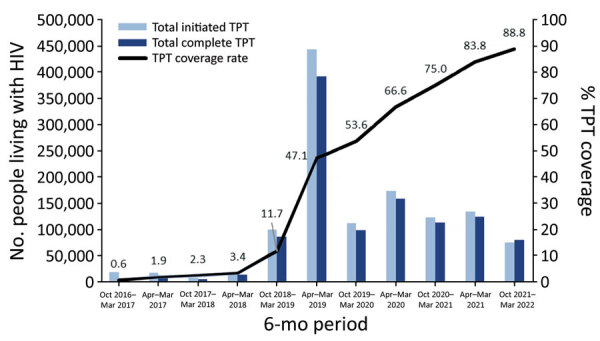
Number of persons initiating and completing tuberculosis preventive therapy among persons living with HIV and overall percentage coverage rate in Uganda, 2016–2022. Numbers are given for 11 semiannual periods; values along line indicate percentage coverage for that period. TPT, tuberculosis preventive therapy

From October 2016–March 2017 through October 2021–March 2022, TPT coverage increased from 0.6% to 88.8% (Figure 2). In all periods, TPT coverage was higher for men. After October 2017–March 2018, TPT coverage increased steadily for both age groups; the largest increases were among PLHIV who were <15 years of age. The median regional TPT coverage during October 2021–March 2022 was 88.0% (IQR 84.5%–92.9%); the lowest rate (68.8%) was in the Karamoja region, and the highest rate (100.0%) was in the West Nile region.

TB notification rates increased from 881.1 cases/100,000 PLHIV during October 2016–March 2017 to 972.5 cases/100,000 PLHIV during October 2021–March 2022 (Table 2). Across all periods, TB notification rates were higher among men and PLHIV who were <15 years of age. During October 2021–March 2022, the highest regional TB notification rate was 1,819.2/100,000 PLHIV in the Karamoja region, and the lowest rate was 665.5/100,000 PLHIV in the Acholi region. The average regional TB notification rate during October 2016–March 2017 was 935.0 (IQR 715.4–1,069.9)/100,000 PLHIV compared with 1,032.72 (IQR 876.7–1,095.2)/100,000 PLHIV during October 2021–March 2022. Across all regions except Karamoja, TB notification rates declined during October 2019–March 2020.

**Table 2:** Tuberculosis notification rates per 100,000 persons living with HIV during semiannual periods, in study of tuberculosis preventive therapy among persons living with HIV, Uganda, October 2016–March 2022*

Semiannual periods	Overall	Patient age			Patient sex	
		<15 y	>15 y		M	F
2016 Oct–2017 Mar†	881.1 (7,850/890,938)	NA	NA		NA	NA
2017 Apr–2017 Sep‡	523.4 (5,198/993,070)	465.6 (290/62,279)	331.2 (3,083/930,791)		642.0 (2,106/328,026)	201.0 (1,267/630,451)
2017 Oct–2018 Mar†	860.1 (8,866/1,030,756)	NA	NA		NA	NA
2018 Apr–2018 Sep‡	1,015.3 (11,186/1,101,716)	1,633.7 (1,035/63,353)	294.1 (3,054/1,038,363)		547.9 (2,128/388,410)	274.9 (1,961/713,306)
2018 Oct–2019 Mar	1,028.3 (11,284/1,097,366)	1,553.2 (929/59,812)	998.0 (10,355/1,037,554)		1,694.1 (6,528/385,331)	667.9 (4,756/712,035)
2019 Apr–2019 Sep	1,094.4 (12,566/1,148,258)	1,607.6 (985/61,273)	1,065.4 (11,581/1,086,985)		1,774.4 (7,195/405,491)	723.1 (5,371/742,767)
2019 Oct–2020 Mar	918.9 (11,037/1,201,166)	1,182.6 (730/61,731)	904.6 (10,307/1,139,435)		1,489.6 (6,336/425,358)	605.9 (4,701/775,808)
2020 Apr–2020 Sep	710.2 (8,650/1,218,006)	756.6 (462/61,062)	707.7 (8,188/1,156,944)		1,148.3 (4,956/431,584)	469.7 (3,694/786,422)
2020 Oct–2021 Mar	866.8 (10,747/1,239,829)	1,144.3 (678/59,249)	854.1 (10,083/1,180,580)		1,356.2 (5,921/436,577)	602.6 (4,840/803,252)
2021 Apr–2021 Sep	908.8 (11,511/1,266,588)	1,175.1 (703/59,925)	895.6 (10,808/1,206,763)		1,388.7 (6,185/445,390)	648.6 (5,326/821,198)
2021 Oct–2022 Mar	972.5 (12,483/1,283,662)	1,416.5 (813/57,396)	951.7 (11,670/1,226,266)		1,504.8 (6,788/451,092)	684.0 (5,695/832,570)

*Values are no./100,000 population (actual number of notifications among PLHIV/total PLHIV population). Tuberculosis notification rate is defined as the number of registered new and relapsed TB cases with documented HIV-positive status during the reporting period divided by the total number of PLHIV currently receiving antiretroviral treatment at PEPFAR-supported sites in Uganda. NA, not applicable; PEPFAR, US President’s Emergency Plan for AIDS Relief. †Total number of tuberculosis cases disaggregated by age and sex were not available. ‡Disaggregated totals for age groups and sex do not equal the overall totals in periods 2 and 4 because of synchronization issues between DATIM (Uganda DATIM version 1.31 MER 2.5, updated January 4, 2021; https://ug.datim4u.org) and the national reporting system; only the sites in which disaggregated totals were consistent with the national reporting system were included.

## Conclusion

In 6 years, Uganda successfully scaled up TPT coverage among a large cohort of PLHIV on ART. Key enablers of success were strong country leadership and ownership, integration of HIV and TB programs, data-driven stakeholder engagement, stable supply chains, and data use for continuous program improvement. However, TB notification rates increased, likely reflecting improvements in TB case reporting as part of efforts to provide TPT to all eligible PLHIV. Over time, TPT completion rates increased, especially during October 2019–March 2020, after the 100-day scale-up campaign (Appendix) (*14*). Although TPT coverage has continued to expand since 2020, negative effects of the COVID-19 pandemic are reflected by declines in TPT initiations and completions during the October 2019–March 2020 and April–September 2020 periods (*3*).

Considering available evidence (*6*–*8*), reducing the burden of TB among PLHIV in Uganda is feasible given high TPT coverage and if the following priorities are continued. First, full TPT coverage should be maintained, including among newly identified eligible PLHIV; shorter TPT regimens should be leveraged for high initiation and completion rates (*15*), and data should be routinely collected for close program monitoring. Second, enhanced TB case reporting is warranted, including among the general population, because the effects of high TPT coverage have not yet translated into declining TB notification rates among PLHIV. Ensuring early identification and treatment initiation for confirmed patients could include quality TB screening, testing, and prompt treatment. Third, newly recommended shorter TB treatment regimens should be considered standards of care to improve patient outcomes.

The first limitation of our study is that discrepancies were observed during some periods between disaggregated and overall totals because the national reporting systems integrated PEPFAR reporting requirements for TB-related data. Second, aggregate patient data are prone to ecologic fallacy and limited our ability to analyze person-level factors affecting TPT initiation and completion. Third, disaggregated data for TB cases during October 2016–March 2017 and October 2017–March 2018 were missing because of changes in data reporting requirements.

In summary, programmatic data indicate that almost all PLHIV in Uganda have received TPT, accelerating progress toward global targets for treatment coverage. Investments in timely TB screening, diagnosis, and earlier treatment during disease course should remain high priorities for TB/HIV prevention programming.

Appendix Additional information for tuberculosis preventive therapy among persons living with HIV, Uganda, 2016–2022.
