# Supplementary material for: Tuberculosis Preventive Therapy among Persons Living with HIV, Uganda, 2016–2022
Source: Emerg Infect Dis. 2023 Mar;29(3):609–13. doi: 10.3201/eid2903.221353 (PMC9973710; doi:10.3201/eid2903.221353)
Supplement: Appendix — Additional information for tuberculosis preventive therapy among persons living with HIV, Uganda, 2016–2022. [file 22-1353-Techapp-s1.pdf]

Article DOI: https://doi.org/10.3201/eid2903.221353

EID cannot ensure accessibility for supplementary materials supplied by authors. Readers who have difficulty accessing supplementary content should contact the authors for assistance.

Tuberculosis Preventive Therapy among Persons Living with HIV, Uganda, 2016–2022

Appendix

In May 2019, Uganda made a benchmark visit to Kenya with the purpose of learning about the scale-up of tuberculosis preventive therapy (TPT) among people living with HIV (PLHIV). Kenya was chosen for this visit given a successful TPT scale-up campaign called 100-days rapid results TPT initiative, which had been concluded. The Uganda team was comprised of staff from the Uganda Ministry of Health (MOH) AIDS Control Program and National TB and Leprosy Program along with PEPFAR (United States President's Emergency Plan for AIDS Relief) technical staff. The Kenya team was comprised of persons from the US Centers for Disease Control and Prevention and Ministry of Health and Sanitation.

As a result of the visit to Kenya, Uganda MOH developed a TPT campaign called the 100-Day Accelerated Isoniazid Preventive Therapy Scale Up Plan. This campaign was launched in July 2019. Under the leadership of Uganda MOH, a national task force for accelerated TPT scale-up was established, with strong representation from funding stakeholders, implementing partners, and civil society. Strategic information partners helped identify priority sites contributing >80% PLHIV to the total number of PLHIV on PEPFAR-supported antiretroviral therapy who were still eligible for TPT. A total of 1,405 sites were identified. Each of these sites was allocated specific weekly TPT enrollment targets.

To monitor achievements throughout the TPT campaign, a weekly reporting dashboard was generated to track enrollment and provide weekly feedback through virtual engagements with regional and district health teams. Tools, including information, communication, and

education materials and job aides, were printed and shared with site level managers to facilitate information and knowledge sharing during mentorships.
